# Iron as a Central Player and Promising Target in Cancer Progression

**DOI:** 10.3390/ijms20020273

**Published:** 2019-01-11

**Authors:** Michaela Jung, Christina Mertens, Elisa Tomat, Bernhard Brüne

**Affiliations:** 1 Institute of Biochemistry I, Faculty of Medicine, Goethe-University Frankfurt, Theodor-Stern-Kai 7, 60590 Frankfurt, Germany; m.jung@biochem.uni-frankfurt.de (M.J.); mertens@biochem.uni-frankfurt.de (C.M.); 2 Department of Chemistry and Biochemistry, University of Arizona, 1306 E. University Blvd., Tucson, AZ 85721-0041, USA; tomat@email.arizona.edu; 3 German Cancer Consortium (DKTK), Partner Site Frankfurt, Theodor-Stern-Kai 7, 60590 Frankfurt, Germany; 4 Project Group Translational Medicine and Pharmacology TMP, Fraunhofer Institute for Molecular Biology and Applied Ecology, 60596 Frankfurt, Germany

**Keywords:** iron homeostasis, lipocalin-2, macrophage polarization, tumor progression, iron chelators

## Abstract

Iron is an essential element for virtually all organisms. On the one hand, it facilitates cell proliferation and growth. On the other hand, iron may be detrimental due to its redox abilities, thereby contributing to free radical formation, which in turn may provoke oxidative stress and DNA damage. Iron also plays a crucial role in tumor progression and metastasis due to its major function in tumor cell survival and reprogramming of the tumor microenvironment. Therefore, pathways of iron acquisition, export, and storage are often perturbed in cancers, suggesting that targeting iron metabolic pathways might represent opportunities towards innovative approaches in cancer treatment. Recent evidence points to a crucial role of tumor-associated macrophages (TAMs) as a source of iron within the tumor microenvironment, implying that specifically targeting the TAM iron pool might add to the efficacy of tumor therapy. Here, we provide a brief summary of tumor cell iron metabolism and updated molecular mechanisms that regulate cellular and systemic iron homeostasis with regard to the development of cancer. Since iron adds to shaping major hallmarks of cancer, we emphasize innovative therapeutic strategies to address the iron pool of tumor cells or cells of the tumor microenvironment for the treatment of cancer.

## 1. Iron and Cancer

Iron is the most abundant element by mass on the Earth and is a growth-limiting factor for virtually all organisms. Considering the poor bioavailability of iron, the interplay of different proteins involved in iron import, storage, and export has to be tightly regulated, as there is no excretory route for excess iron. The ability of iron to get oxidized or reduced enables iron to take part in free radical generating reactions. Among them is the Fenton reaction; ferrous iron donates an electron to hydrogen peroxide to produce the hydroxyl radical, a highly reactive oxygen species. As a result, iron is potentially mutagenic by causing DNA strand breaks, which provokes cellular transformations, or induces protein as well as lipid modifications within malignant cells. In turn, this may cause a more aggressive tumor cell behavior [1,2,3]. Accumulation of iron-dependent lipid modifications promotes a specific form of cell death, known as ferroptosis [4]. This is a distinct type of cell death compared to apoptosis, necrosis, or autophagy [5]. During ferroptosis, a lethal metabolic imbalance arises from glutathione depletion, which inactivates the lipid repair enzyme glutathione-dependent peroxidase 4, GPX4, with the subsequent accumulation of lipid peroxides [6]. Nevertheless, mammalian cells require sufficient amounts of iron to satisfy metabolic needs and accomplish specialized functions. Only to mention a few, DNA polymerases and helicases contain iron-sulfur groups that rely on iron as essential co-factors [7]. In addition, cellular iron availability not only controls mitochondrial respiration, but also affects citric acid cycle enzymes [8,9].

The malignant cancer phenotype is often found in association with a deregulated iron homeostasis, particularly the expression of iron-regulated genes that fuel their higher metabolic iron demands needed for division, growth, and survival [10]. This surplus of iron is needed not only both during early steps of tumor development, e.g., enhanced survival [11] and proliferation of transformed cells [12], but also during late stages to promote the metastatic cascade. Here, iron is crucial in remodeling the extracellular matrix and increasing the motility and invasion of cancer cells [13]. A dysfunctional or deregulated iron metabolism in cancer patients often results in a decreased red blood cell (RBC) count [14] and anemia is detected in approximately 40–70% of all cancer patients [15,16]. Furthermore, cancer-induced anemia as well as inflammation-associated anemia is characterized by reduced erythropoiesis [17]. Mechanistically, cancer-induced anemia is caused by the secretion of inflammatory factors, such as tumor necrosis factor (TNF)-α and interleukin-6 (IL-6). They inhibit erythropoiesis due to iron restriction in the reticuloendothelial system [18]. The role of iron in cancer progression was also documented by experimental animal models [19,20,21]. Mice fed a low-iron diet prior to implanting tumor cells showed significantly delayed tumor growth [22]. In addition, staining iron deposits, it became apparent that tumors outcompete the natural iron reservoirs in liver and spleen [23]. As a result, RBC recycling and iron storage in liver and spleen are compromised and erythropoiesis is significantly reduced, finally provoking anemia. A recent study from Theurl and colleagues provides a sophisticated study of RBC clearance and iron recycling in a mouse model [24]. Senescent RBCs are taken up by monocytes in the bloodstream and transported to both liver and spleen. Interestingly, accumulation of iron-recycling macrophages was exclusively observed in the liver, thus identifying the liver as the main organ for RBC recycling. These findings are of crucial importance since the recycling of senescent RBC by reticuloendothelial macrophages is of utmost importance to recover iron in order to ensure efficient erythropoiesis.

A growing number of studies explore the role of iron-related proteins in the context of cancer. Apparently, the expression of different iron-regulated genes such as the transferrin receptor (TfR1) [25], ferritin light chain (FTL) [26,27], and the iron regulatory protein (IRP)-2 [28] in tumor cells correlated with a poor prognosis, a higher tumor grade, and increased chemoresistance. Given the complex network of iron regulatory genes in cancer cells and their role for tumor growth and survival, a better understanding of their regulation and interplay is warranted. The identification of recently acknowledged new iron-regulated genes, such as lipocalin-2 (Lcn-2) as well as siderophore-binding proteins, might help to understand how the tumor exploits systemic and local iron management.

## 2. Iron Metabolism in Cancer Cells

Circulating iron is normally found complexed in transferrin (Tf), circulating in the bloodstream. Tf is taken up in peripheral tissue by binding to the TfR1. The ligand–receptor complex is endocytosed and recycled within the endosome, releasing ferric iron, which is exported into the cytosol by the divalent metal transporter (DMT)-1. High TfR1 expression correlated not only with a reduced response to chemotherapy [25], but also increased phosphorylation of src in breast cancer, promoting tumor cell division, motility, and adhesion. Moreover, the homologous TfR2 is also frequently upregulated in cancer cells [29,30]. Therefore, the Tf/TfR system not only enhances iron uptake, but also provokes tumor cell survival [31]. Ultimately, tumor cells adjust intracellular iron metabolism to favor iron accumulation, by increasing iron uptake and storage, at the same time decreasing iron export. Imported iron enters the bioactive labile iron pool (LIP), which provides it for metabolic and proliferative purposes. The amount of the LIP is sensed by post-transcriptional mechanisms of cytosolic iron-regulated RNA binding proteins 1 and 2 (IRP-1 and IRP-2) to fine-tune uptake, storage, and release of iron. When intracellular iron is low, IRPs interact with conserved iron-responsive elements (IREs) in the untranslated regions (UTRs) of central genes accounting for iron homeostasis [32,33]. Binding of IRPs to IREs in the 5’-UTR attenuates translation, whereas binding to IREs in the 3’-UTR stabilizes respective mRNAs and fosters translation. Thus, when iron is high, mRNAs of TfR and DMT-1 are unstable and get degraded, which ultimately decreases iron uptake and transport. Simultaneously, iron storage is enhanced by releasing the translational blockade of ferritin (FT) heavy chain (FTH) and FTL. Thereby, excess iron that is not utilized, can be stored in the iron storage protein FT and can later be accessed by ferritinophagy [34]. Breast cancer cells with a more aggressive mesenchymal phenotype display higher amounts of intracellular FT. Moreover, serum concentrations of FT are increased in cancer patients compared to healthy individuals [35]. For this reason, the expression of FT is considered a prognostic marker for some cancer subtypes such as squamous cell carcinoma or breast cancer [27,36,37]. Interestingly, extracellular FT stimulates proliferation of breast cancer cells independently of the iron content, acting as an inflammatory effector mechanism to directly support tumorigenesis [38,39,40].

**Figure 1:**
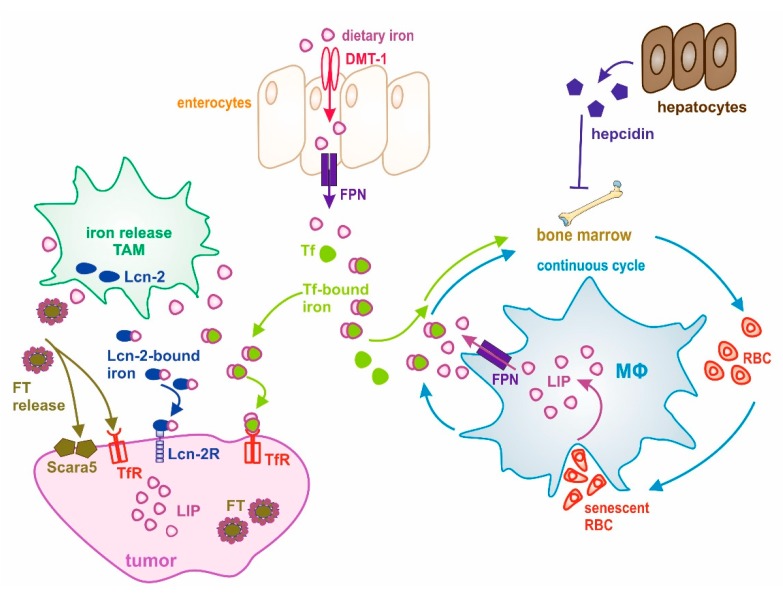
Schematic overview of the interplay between systemic and local iron homeostasis in the tumor. Dietary iron is absorbed by enterocytes through divalent metal transporter 1 (DMT-1) and released to the circulation through the iron exporter ferroportin (FPN). After its release, iron is rapidly loaded onto transferrin (Tf) for systemic transport. Macrophages (MΦ) take a central role in maintaining systemic iron homeostasis, which is accomplished through a continuous cycle of iron recycling from senescent red blood cells (RBCs). Iron then joins the labile iron pool (LIP) and is then donated to the circulation, where it is bound to Tf for its transport to cells and tissues having a need for iron or to the liver, where iron is stored. Systemic iron homeostasis is controlled by the expression of hepcidin from hepatocytes. During cancer, this cycle is deregulated and systemic iron availability is decreased through its sequestration within MΦ, finally causing anemia. At the tumor site, tumor-associated macrophages (TAM) adopt an iron-release phenotype and donate iron to the microenvironment. Iron can be released via FPN and loaded onto Tf for its uptake by cancer cells via the Tf receptor (TfR). Alternative iron donation pathways have evolved: (1) lipocalin-2 (Lcn-2)-bound iron is taken up by its high-affinity receptor Lcn-2R, and (2) macrophage-released ferritin (FT) might be taken up through Scara5 (FTL) or TfR (FTH) by tumor cells.

Iron, when neither metabolically used nor stored in FT, is exported from cells to the circulation by the iron exporter ferroportin (FPN), gets oxidized by ceruloplasmin or hephaestin, and is loaded to Tf. The FPN efflux system represents one of the key mechanisms to adjust the iron amount in the body and to affect the ratio of stored and released iron [41]. In invasive tumor areas, iron export via FPN is lower compared to normal tissue, with the notion that FPN expression in carcinomas inversely correlated with patient survival and disease outcome [42]. A decreased FPN expression in tumor cells is associated with enhanced availability of LIP-associated iron in cultured breast cancer cells. This effect increased tumor growth in a breast cancer xenograft model, correlating with the aggressiveness of breast cancer subtypes. Consistently, an increased expression of FPN in tumor cells correlated with better patient outcome, as does the absence of estrogen receptor, low histological grade, or a low grade of lymph metastasis. The expression of FPN is regulated by the acute-phase protein hepcidin, which induces internalization and degradation of FPN upon its binding, thereby attenuating the iron export capacity [43]. Peripheral tissues also secrete hepcidin, which, unlike liver-secreted hepcidin, is thought to act locally. Tissue sequestration and systemic iron levels are regulated by hepcidin, by controlling FPN-facilitated iron release into the plasma of all cells that handle iron, including intestinal enterocytes, hepatocytes, and macrophages [44]. Levels of hepcidin are increased during inflammation and decreased by iron insufficiency and erythropoiesis [45,46,47]. In cancer patients, hepcidin levels are often elevated, likely because of cancer driven inflammation [48,49]. Enhanced hepcidin expression in breast cancer patients points to an autocrine/paracrine regulatory mechanism in order to reduce control local tumor iron efflux [50]. A scheme describing how tumors exploit physiological iron resources is given in Figure 1.

## 3. Iron in the Tumor Microenvironment—Role of Tumor-Associated Macrophages

The role of iron for cancer development is tightly linked to its ability to modulate innate and adaptive immune responses of macrophages or T cells. In order to control iron availability, immune cells adapt their phenotype accordingly to defend the host against invading pathogens. Since tumor cells might be recognized as foreign in the first place, it is not surprising that immune cells get polarized to adjust the iron metabolism at the systemic as well as local tumor levels [51,52,53]. As a consequence, tumor cells compete for iron with immune cells of their local microenvironment. The inflammatory nature of the tumor microenvironment and the presence of inflammatory stimuli are critical regulators of iron availability. During early stages of carcinogenesis, pro-inflammatory cytokines endorse iron sequestration in macrophages and enhance the production of reactive oxygen species as a first-line anti-tumor defense. However, chronic inflammation or smoldering inflammation often creates an equilibrium between killing of immunogenic tumor cells and immune tolerance, finally driving tumor outgrowth. Outgrowth is supported as tumor cells often evade immune recognition or even acquire an immunosuppressive phenotype [54]. Based on the intimate interplay of tumor cells and tumor-infiltrating immune cells, the latter ones are educated to a tumor-supportive, anti-inflammatory phenotype that significantly promotes tumor neovascularization, metastasis, growth and survival [55]. In contrast to the iron sequestration phenotype of inflammatory macrophages induced by pro-inflammatory cytokines and danger-associated molecular patterns (DAMPs), anti-inflammatory macrophages and lymphocytes show an iron release phenotype, donating and distributing iron within the tumor microenvironment. These observations were recently underlined by Marques et al., describing an “iron utilization” phenotype of tumor cells, whereas tumor-infiltrating macrophages and lymphocytes represented an “iron donor” phenotype [50]. In addition, macrophages are capable of ferritin secretion, whereby tumor growth is promoted [40]. These findings were corroborated by a variety of studies showing ferritin expression mainly in the stromal compartment of tumor tissue [26,27]. Of note, the appearance of tumor-supporting, iron-donating immune cells, in particular macrophages, is associated with tumor size and aggressiveness as well as poor patient prognosis [56,57].

Since macrophages are characterized by high functional plasticity and heterogeneity of activation, it is speculated that distinct macrophage subpopulations are found within the same tumor, with the individual phenotypes coined by their localization and microenvironmental stimuli [58]. Therefore, it seems appropriate to consider polarization of the TAM as a continuum of functional activation phenotypes [59,60,61,62,63] rather than distinct subpopulations. Consequently, some macrophage polarization states might support, whereas others antagonize tumor cells. These observations also hold true for the iron-regulated gene signature of distinct macrophage phenotypes. The profile can be characterized by expression of a particular subset of iron-regulated genes to take up iron, store it, or export it in order to donate it to neighboring cells. Pro-inflammatory macrophages are prone to iron retention. They display an iron sequestering phenotype characterized by enhanced iron uptake and storage, but attenuated release [64,65,66,67]. In contrast, anti-inflammatory macrophages are predisposed to iron export and the distribution of iron to the extracellular space, whereas iron storage is reduced. As alternatively activated macrophages scavenge senescent and/or apoptotic cells [68], they play an important role in tissue repair, regeneration, resolution of inflammation, and iron recycling. Consequently, they show a high expression of scavenger receptors such as CD163 and CD91. This allows for the uptake of iron-containing heme clusters, which in turn enhances the expression of heme oxygenase 1 (HO-1) [69]. The iron-release phenotype is associated with upregulation of the iron exporter FPN, while the iron storage protein FT is downregulated [67]. The majority of the heme-recycled iron joins the LIP, with only a small proportion actually being stored in FT. Non-heme-bound iron can be taken up through the DMT-1. These features allow anti-inflammatory macrophages to rapidly mobilize and redistribute iron to the local microenvironment in order to support the demand of surrounding cells. This iron-donating macrophage phenotype was directly linked to enhanced tumor cell proliferation and growth, both in vitro and in vivo [64,66,67,70,71,72].

Recently, we provided evidence that TAM adopt an iron-release phenotype due to their interaction with dying tumor cells, whereby iron availability was increased within the tumor microenvironment [66]. Under these conditions, TAM expressed higher levels of the high-affinity iron-binding protein lipocalin-2 (Lcn-2). Lcn-2 turned out to export iron from TAM, while depletion of the established iron exporter FPN did not alter their iron release capacity [66]. These observations suggest the existence of an alternative iron transport pathway in the tumor microenvironment, operating independently of FPN. The inability of FPN to add to iron export under these conditions might be the local expression of hepcidin, which compromises its expression. Interestingly, large amounts of hepcidin are found both within the tumor microenvironment and systemically in cancer patients [73,74,75,76]. However, recent findings suggest that excess amounts of heme or iron in hemorrhagic tumor areas are detrimental to the macrophage iron phenotype by shifting the anti-inflammatory, pro-tumor macrophage phenotype to a pro-inflammatory and thus anti-tumoral one [77]. These observations are somehow mirrored when macrophages are exposed to hemolytic RBCs during e.g., sickle cell anemia [78]. Based on the heme-induced polarization shift, macrophages acquire a more pro-inflammatory phenotype and exacerbate tissue damage [78]. Additional studies support the idea that excess iron predispose macrophages towards a pro-inflammatory phenotype [79,80], thereby linking macrophage iron handling to their role in inflammatory disease.

Since tumors demand an excess of iron, both during early steps of tumor development and late metastatic processes, recent paradigms of macrophage iron polarization are of clinical interest. The fact that TAMs actively release iron to the tumor microenvironment positions them at the center of pathways associated with the concepts established as the “hallmarks of cancer”. Further investigations and mechanistic insights regarding the development of TAM heterogeneity and functional iron plasticity are urgently needed.

## 4. Strategies to Target Iron Trafficking in the Tumor Microenvironment

Iron handling in the tumor microenvironment emerged as an important aspect of tumorigenesis. Since hepcidin arises as a key iron-regulated gene due to its function as a sensor for systemic iron availability, several strategies are envisioned to manipulate its expression [81]. First, pharmacological approaches used neutralizing antibodies or aptamers to directly target hepcidin. Other ways to control hepcidin expression are based on inhibition of bone morphogenic proteins (BMPs)/SMAD as well as IL-6/signal transducers and activators of transcription (STAT) 3 signaling pathways [82,83]. Interfering with IL-6 signaling using tocilizumab, an antibody directed against the IL-6 receptor was effective in treating chronic anemia associated with arthritis [84]. First promising anti-cancer efficacy was recently provided by the simultaneous inhibition of the IL-6 receptor using tocilizumab and the IL-8 receptor by reparixin, showing significantly decreased metastasis of breast cancer cells to the lung, liver, and lymph nodes in a mouse mammary xenograft model [85]. However, no direct correlation of synergistically blocking the IL-6 and IL-8 receptors and hepcidin expression or the effect on cancer-related anemia was reported in this study. However, it seems that blocking hepcidin through manipulation of the IL-6 signaling cascade is feasible for the treatment of cancer anemia.

Current approaches aim at reactivating iron release from the reticuloendothelial system, which is compromised during tumor development and manifested as anemia in cancer patients. As prospective clinical data are still missing, the approach to targeting hepcidin for cancer treatment is still not fully accepted. The same holds true for directly neutralizing hepcidin using antibodies. Many of the currently available drugs and/or antibodies interfering with hepcidin expression or activation show promising effects in the treatment of chronic anemia, which is also found in cancer patients. Due to a lack of long-term follow-up studies in cancer patients, it is difficult to predict both the efficacy and safety of those drugs in cancer treatment. There are also attempts to develop FPN stabilizers in order to reactivate iron efflux form tumor cells [86,87,88]. As pathways regulating the hepcidin-FPN axis are complex, the majority of present pharmacological strategies aim at acutely interfering with protein expression and/or activity. Cancer cells reprogram their iron metabolism to increase net iron influx. This is accomplished by upregulating proteins for iron uptake such as Tf. The Tf/TfR system represents one of the major routes for iron acquisition, both in normal and malignant cells [89]. Upregulation of this highly conserved iron acquisition pathway is found in a variety of cancers, including breast and colon [90,91,92,93]. Thus, it appears logic to apply an anti-TfR strategy as a therapeutic measure. Roughly 30 years ago, initial studies were conducted to explore the anti-neoplastic capacity of anti-TfR monoclonal antibodies [94,95,96]. Phase I trials provided promising results, without any indication of major side effects in patients [97,98]. However, treatment was only effective in some cancers such as adult T-cell leukemia/lymphoma (ATL) and leukemia [99,100]. Further studies revealed that anti-TfR treatment was specifically encouraging for therapeutic approaches in hematologic cancer due to the fact that cells of the hematopoietic lineage are highly iron-dependent. At the same time, these observations raised major concerns for the use of anti-TfR antibodies for the treatment of other tumor types. The problem may arise that maturing erythroid cells would severely be affected by anti-TfR antibodies, which, in turn, may disturb erythropoiesis and cause anemia. Taking the upregulation of the TfR on the tumor cell surface into account, a Trojan horse strategy was tested to shuttle therapeutic molecules into malignant cells. In fact, a variety of studies confirmed an improved anti-cancer drug uptake upon conjugation to a TfR monoclonal antibody [101,102,103]. Not only to mention that TfR expression is significantly upregulated on cancer cells, it also represents a very effective receptor-mediated endocytosis system. Therefore, the Tf–TfR system is considered a promising target to enhance the uptake of drugs that are specifically conjugated to Tf to be recognized by the TfR and to facilitate uptake in, i.e. multidrug-resistant tumor cells [104]. The exceptional capacity to transport drugs across the blood–brain barrier makes the Tf–TfR axis valuable for the treatment of brain tumors through TfR-mediated transcytosis [105]. As the TfR is expressed at high levels at the blood–brain barrier [106,107] due to its physiological function to deliver iron to the brain, it is one of the most studied molecules for receptor-mediated drug delivery to the brain. Using either Tf or TfR as targeting moieties, current studies with both direct conjugation and immunotoxins focus mainly on the development of treatment approaches against brain tumors [108,109,110,111,112]. Another consideration is the uptake of secreted FT by the TfR [113]. Since FT assembles into spherical cage-like structures and has the potential to reversibly disassemble within cells, it represents an attractive target for nanostructure research in the cancer context [114]. Such naturally occurring structures are advantageous over synthetically ones due to their low toxicity and negligible immune responses. FT nanocages are used to encapsulate chemotherapeutic agents such as doxorubicin [115] or gold ions to induce tumor cell death [116].

It needs also consideration that cancer cells might have evolved alternative strategies to take up iron trough hitherto-unappreciated transport proteins. Lcn-2 might accomplish such a role due to its ability to scavenge iron-loaded siderophores [117,118]. Siderophores are small, low-molecular-mass iron-binding ligands known from iron acquisition mechanisms used by bacteria [119]. Interestingly, also higher organisms such as fungi or mammals are able of producing this type of iron-scavenging molecules [120,121,122]. Lcn-2 shows an extraordinary high affinity to bind iron-loaded catecholate-type siderophores. However, it still remains unclear if siderophores are indeed produced in mammals to mediate Lcn-2–iron binding or if Lcn-2 takes advantage of bacterial siderophores, residing within mammals from e.g., commensal bacteria. A recent study proposes that bacterial siderophores do not only serve as iron scavengers to limit bacterial growth, but are also able to support the host’s iron homeostasis [123]. Binding to the α subunit of ATP synthase, mitochondrial iron uptake is promoted by enterobactin, secreted from commensal bacteria. These features have to be considered with regard to the iron-transporting function of Lcn-2, taking its extraordinary function in innate immunity into account. Another aspect of Lcn-2 during infectious diseases is its ability to control neutrophil function [124,125]. Neutrophils from Lcn-2^–/–^ animals do not migrate to sites of infection and do no longer respond to chemotactic stimuli [124], which however can be reverted by the addition of recombinant Lcn-2 [125].

Lcn-2 is recognized and internalized by cells via their highly expressed high-affinity Lcn-2 receptor (Lcn-2R) and/or the low-affinity megalin receptor. Several studies in humans suggest Lcn-2 as a pro-tumorigenic factor in breast cancer, correlating with a decreased survival and reduced responsiveness to neoadjuvant chemotherapy [126,127]. It has been observed that human breast tumors contain enhanced amounts of Lcn-2, especially during advanced stages [128]. Consistently, elevated Lcn-2 levels in the urine of breast cancer patients are correlated with a poor metastatic outcome [129]. Additionally, experimental transgenic tumor-bearing mouse mammary tumor virus (PyMT) mice exhibit higher Lcn-2 plasma levels compared to controls, and Lcn-2-deficient PyMT mice developed fewer tumors than Lcn-2-competent littermates [130]. Corroborating the role of Lcn-2, expression of Lcn-2R is also indicative for a poor prognosis, and a reduced survival rate but increased invasion in several cancer entities [131,132]. These observations strengthen the concept that Lcn-2 promotes its pro-tumor functions via Lcn-2R signaling. Lcn-2 was also shown to induce epithelial-to-mesenchymal transition (EMT) through upregulation of the EMT-associated transcription factors Snail1, Slug, and Twist1, which, in turn, influence the expression of epithelial and mesenchymal markers to promote invasiveness. While the majority of studies so far focused on mechanisms promoted by tumor cell-derived Lcn-2, recent data from our group suggest that stromal Lcn-2 promoted metastasis by enhancing EMT and lymphangiogenesis [133]. Moreover, we proposed that iron might be the missing link to understand the tumor-promoting role of Lcn-2. In fact, it was speculated that the iron load of Lcn-2 defines pro-tumor characteristics of Lcn-2. However, it is still unknown how tumor cells selectively take up iron-loaded Lcn-2 relative to iron-free Lcn-2 or how the latter is antagonized within tumor cells in order to avoid its reported apoptotic effects.

## 5. Novel Iron Chelation-Based Treatment Strategies

Cancer cells have a higher demand for iron in order to sustain their proliferative capacity. Considering this as their Achilles’ heel, several approaches seek to interfere with iron handling in cancer cells, either by directly modulating iron-regulated genes or by using iron chelators. The anti-neoplastic potential of iron chelators is accomplished, at least in part, by inhibiting DNA synthesis, causing a G1-S-phase cell cycle arrest, attenuating EMT, correcting epigenetic signatures of malignant tumor cells, and/or promoting cancer cell apoptosis. For instance, the natural siderophore desferrioxamine (DFO, Desferal^®^) [134,135] as well as the synthetic chelator derasirox (DFX, Exiade), which are clinically employed to treat iron overload due to chronic blood transfusion therapy [134], and the thiosemicarbazone Triapine^®^ [136,137] have shown promising anti-cancer activity in clinical trials [138]. Unfortunately, due to serious side effects in several cases, e.g., hearing abnormalities, nephropathy, optic neuropathy, and growth failure in children, their applicability during cancer treatment is hampered [135,139]. While most studies aim at targeting iron in tumor cells, approaches to addressing iron chelation in cells of the tumor microenvironment is still lacking. Generally, employing metal chelators in the context of cancer treatment remains challenging, when compared to their applications for systemic iron depletion. Fundamental to successfully approach chelation therapy in cancer will be the capacity to target iron selectively in malignant cells without affecting normal tissue or the extracellular space. Chelators derived from bacterial siderophores (e.g., DFO) or other high-affinity scaffolds (e.g., Triapine) lack the required selectivity and thus are not designed to target specifically the higher iron demand of malignant cancer cells, which generally show normal or low systemic iron levels. Moreover, the high hydrophilicity and unfavorable pharmacokinetics of several chelators do not allow reaching effective intratumoral concentrations [59]. To date, none of the iron chelators employed in the clinic for iron overload disorders has obtained approval for the cancer setting. More recently, high-throughput screening identified an iron chelating agent as a promising drug candidate for colon cancer (VLX600) [140], which entered a Phase I clinical trial (identifier: NCT02222363) for refractory advanced solid tumors. A summary of pre-clinical studies and clinical trials that target tumor iron metabolism is given in Table 1.

Prodrug strategies are currently under investigation in order to implement specific prodrug activation/action [152]. The use of disulfide bonds as the activation switch of iron prochelators showed encouraging results in vitro, as iron coordination only occurs following intracellular disulfide reduction, leading to the formation of high-affinity tridentate thiolate chelators [153]. It can be envisioned that a higher reducing environment in cancer cells, compared to the surrounding tissue, makes reductive activation an attractive strategy to enhance cancer cell specificity [153,154], owing to higher amounts of reduced glutathione compared to healthy, neighboring cells [69]. Because of the stability of disulfide linkages in blood plasma, an advantage of the prochelator strategy is their potential supplementation at high doses, which may foster their uptake by tumor cells, while avoiding unwanted side effects in plasma. Disulfide linkers can also be employed to attach tumor-targeting units aimed at imparting higher cancer selectivity. Supported by the widespread clinical use of radiolabeled glucose analogs for tumor visualization by positron emission tomography (PET) [155], glycoconjugation is undergoing intense scrutiny as a method to increase specificity of anti-proliferative agents [156]. Akam et al. explored glucose-conjugated thiosemicarbazone prochelators in colon carcinoma [157], proving enhanced prochelator accumulation in malignant cells. These approaches exploit overexpression of the glucose transporter GLUT1 in a large fraction of cancer phenotypes [158]. Glucose conjugates of several cancer chemotherapeutics, including paclitaxel and doxorubicin [159,160,161], experienced a higher therapeutic efficacy in vivo. These strategies may also be exerted to achieve selectivity in targeting the iron pool of tumor cells.

While most of the current chelator-based intervention strategies focus on tumor cells, targeting iron in the tumor microenvironment has not been explored significantly. Considering that macrophages constitute a major infiltrate in solid tumors and taking their pivotal role in iron metabolism into account, these immune cells are likely a worthwhile target in anti-cancer strategies. We employed the disulfide-based prochelator (TC3-S)_2_ [72] to explore how iron sequestration in macrophages affects their iron homeostasis. Upon intracellular reduction, (TC3-S)_2_ is converted to the thiol chelator TC3-SH, which re-programmed macrophages from an iron release to and iron sequestration phenotype [72]. Future in vivo studies using this approach should address the potentially impactful possibility to interfere with tumor progression by using macrophage-targeted chelation strategies.

Another recent possibility to target excess iron in tumor cells is through induction of ferroptosis, an iron-dependent and peroxidation-driven form of cell death. This type of cell death was recently shown to be executed via the tumor suppressor p53 [162,163], suggesting a natural anti-tumor role of ferroptosis. Interestingly, especially therapy-resistant and drug-tolerant tumor cells are prone to ferroptosis [164,165]. Thus, it might be speculated that inducers of ferroptosis emerge as novel and rather selective anti-cancer drugs.

## Figures and Tables

**Table 1:** Summary of pre-clinical studies and clinical trials that target tumor iron metabolism.

Target	Substance	Outcome	Reference
**TfR**	Monoclonal antibody against tfr	Blocked ex vivo proliferation of malignant T cells from adult T-cell leukemia/lymphoma	[100]
**TfR**	Monoclonal antibody against tfr	Well tolerated in advanced refractory cancer patients, but no partial nor complete remissions	[97]
**TfR**	Monoclonal antibody against tfr	Peripheral blood blasts derived from patients with acute myeloid leukaemia (AML) showed a very heterogeneous tfr expression; tfr expression was related to blast. Proliferative capacity	[99]
**TfR**	Monoclonal antibody against tfr	Antibody treatment inhibits Tf uptake and causes growth inhibition by iron deprivation in malignant human hemopoietic cells	[98]
**TfR**	Liposomal-drug functionalized with transferrin	Enhanced drug targeting to tumors with increased tfr expression; Phase II: gastric, oesophageal adenocarcinoma Phases I and II: solid tumors	NCT00964080 NCT00355888 NCT02354547 NCT02340156 NCT00470613 NCT01517464
**Hepcidin/FPN**	Spiegelmer NOX-H94 (Lexaptepid Pegol)	Inhibits hepcidin-induced FPN degradation in a murine macrophage cell line	[141]
**Hepcidin/FPN**	Spiegelmer NOX-H94 (Lexaptepid Pegol)	Phases I and II: treatment of anemia of chronic disease in cancer patients	NCT01691040 NCT01372137
**Hepcidin /IL-6**	Siltuximab	Improves cancer patient anemia	[142,143]
**Hepcidin /IL-6**	Tocilizumab	Reduction of serum hepcidin and correction of anemia after 6–12 months treatment	[84]
**Tam**	Iron oxide nanoparticles	Tumors injected with iron oxide nanoparticles had significantly smaller tumor sizes	[77,144]
**Iron**	Desferrioxamine (DFO)	Anti-neoplastic activities in leukemia	[145,146]
**Iron**	Desferrioxamine (DFO)	Only partial response in refractory/advanced neuroblastoma patients	[34,147,148,149]
**Iron**	Triapine	Stops tumor growth by blocking cell growth; tested in almost 30 clinical trials, either alone or in combination with other drugs such as cisplatin and gemcitabine	Reviewed in [150]
**Iron**	Deferasirox (EXJADE^®^)	Complete remission of acute monocytic leukemia in a patient;	[151]
**Iron**	Deferasirox (EXJADE^®^)	Phase I: hematologic malignancies	NCT01273766 NCT02233504
**Iron**	Vlx600	Phase I: colon cancer	NCT02222363
**Iron**	Dpc	Thiosemicarbazone chelator; phase I: advanced solid tumors	NCT02688101

## Abbreviations

DMT-1	divalent metal transporter 1
EMT	epithelial-to-mesenchymal transition
FPN	ferroportin
FT	ferritin
FTH	ferritin heavy chain
FTL	ferritin light chain
IRE	iron responsive elements
IRP	iron regulatory proteins
LIP	labile iron pool
Lcn-2	lipocalin-2
PET	positron emission tomography
RBC	red blood cell
UTR	untranslated region
TAM	tumor-associated macrophages
Tf	transferrin
TfR	transferrin receptor
